# Reproducibility of Static and Dynamic Postural Control Measurement in Adolescent Athletes with Back Pain

**DOI:** 10.1155/2018/8438350

**Published:** 2018-07-02

**Authors:** Edem Korkor Appiah-Dwomoh, Steffen Müller, Frank Mayer

**Affiliations:** ^1^Department of Sports and Health Sciences, Clinical Exercise Science, University Outpatient Clinic, University of Potsdam, 14469 Potsdam, Brandenburg, Germany; ^2^University Outpatient Clinic Potsdam, University of Potsdam, 14469 Potsdam, Brandenburg, Germany; ^3^Trier University of Applied Science, Department of Computer Science/Therapy Science, 54293 Trier, Germany

## Abstract

Static (one-legged stance) and dynamic (star excursion balance) postural control tests were performed by 14 adolescent athletes with and 17 without back pain to determine reproducibility. The total displacement, mediolateral and anterior-posterior displacements of the centre of pressure in mm for the static, and the normalized and composite reach distances for the dynamic tests were analysed. Intraclass correlation coefficients, 95% confidence intervals, and a Bland-Altman analysis were calculated for reproducibility. Intraclass correlation coefficients for subjects with (0.54 to 0.65), (0.61 to 0.69) and without (0.45 to 0.49), (0.52 to 0.60) back pain were obtained on the static test for right and left legs, respectively. Likewise, (0.79 to 0.88), (0.75 to 0.93) for subjects with and (0.61 to 0.82), (0.60 to 0.85) for those without back pain were obtained on the dynamic test for the right and left legs, respectively. Systematic bias was not observed between test and retest of subjects on both static and dynamic tests. The one-legged stance and star excursion balance tests have fair to excellent reliabilities on measures of postural control in adolescent athletes with and without back pain. They can be used as measures of postural control in adolescent athletes with and without back pain.

## 1. Background 

Studies suggest that back pain (BP) is a problem in the general and athletic populations [1–5]. It causes disruption of postural control (PC) [6] and alteration in trunk muscle activity [7–9]. Hence, there is a need for periodic assessment and monitoring to identify and appropriately rehabilitate the altered or impaired trunk and postural control. This can be done statically by assessing deviations in the location of the centre of pressure (COP) through measures derived from force plate data using the one-legged stance test [9, 10]. Dynamically, the assessment can be made by completing a movement task whilst maintaining a stable base of support using the star excursion balance test (SEBT) [11].

In typically developing children, the reliability of sway parameters on a force platform using the one-legged stance test are generally moderate to excellent [12]. Healthy young adults also show excellent intra- and intersession reliability [13, 14]. In the only published literature on the reliability of static postural control in athletes, Harringe et al. [15] used a double-leg stance test. However, superior balance is reported in athletes due to repetitive training [16], and a one-legged stance is often required to switch from two legs to one during the performance of sports. Hence, a more challenging task like the one-legged stance test would be appropriate as a static measure for this group of individuals. In the dynamic test, SEBT, Kinzey and Armstrong [11] were the frst to examine the reliability, conducting their study in a healthy general population of adults. They reported moderate to high reliability with intraclass correlation coefficients (ICC) ranging from 0.67 in the right anterior direction to 0.87 in the left anterior and left posterior directions [11]. In adult recreational athletes, Munro & Herrington [17] also reported excellent reliability (ICC; 0.84 to 0.92) for all directions on the test. ICC ranging from 0.84 to 0.87 and 0.51 to 0.93 for the 3 reach directions of the SEBT has also been reported for high school basketball players [18] and primary school children [19], respectively. The SEBT has been used to assess postural control in individuals with back pain, athletes [20] and nonathletes alike [21] without first establishing the reliability of the test in these groups. Also, in published literature, measurement of the reliabilities of both the one-legged stance test and the SEBT took place in healthy subjects; therefore it cannot be assumed that this will remain the same in injured individuals and athletes and different age groups. In addition to this, athletes have superior balance abilities due to training [16], and this needs to be taken into consideration. Furthermore, BP damages the sensory tissues and pain inhibition in the lumbar spine and trunk is believed to affect the PC mechanism [7, 22]. This might lead to the adoption of alternative PC strategies to cope with the new demands introduced by pain [10]. Also, individuals with BP show changes in the position of their centre of pressure compared to pain-free subjects [23, 24], and differences in PC exist between injured and noninjured individuals [15]. Therefore, the aim of the study was to determine the test-retest reproducibility of static and dynamic PC measurements in adolescent athletes with and without BP using the one-legged stance for the former and the SEBT for the latter. A second aim was to determine whether reproducibility of these tests was different between adolescent athletes with and without BP.

## 2. Methods

### 2.1. Participants

A total of 35 adolescent athletes were recruited for the study. 4 subjects (1 BP and 3 NBP) were excluded due to the report of chest pains and knee and shin injuries prior to retest as well as data acquisition challenges. Therefore, 14 BP athletes (14.6 ± 1.4 years, 66.0 ± 8.3 kg, 173.8 ± 5.3 cm, 4.7 ±2.5 training years, 8.9 ± 3.9 training sessions/week, 96.1 ± 18.0 training minutes/session) and 17 NBP athletes (13.8 ±1.5 years, 58.8 ± 13.2 kg, 170.3 ± 12.2 cm, 3.9 ± 2.5 training years, 7.1 ± 3.3 training sessions/week, 98.8 ± 23.2 training minutes/session) were included in the final data analysis. The athletes were from 7 different sport disciplines: athletics (n = 6), rowing (n =7), canoeing (n = 4), swimming (n = 1), football (n = 8), handball (n = 3), and volleyball (n = 2). A pain questionnaire consisting of a numeric rating scale of 1 (no pain) to 5 (severest pain) in the form of smiley faces was used to determine participants with BP [25]. BP was not confined to a specific region of the back and was defined as pain rating from 2 to 5 on the pain questionnaire. The mean BP score at initial testing was 3 ± 0.8 and 2.8 ± 1.0 at retest. Subjects with lower and upper limb injuries, head injuries, vision problems, and any other complaints that could have affected the balance measurement were excluded. The institution's ethics committee gave ethical approval and participants and their parents or guardians gave written informed consent before data collection.

### 2.2. Procedure

Age, gender, weight, height, number of training years, training days per week, training minutes per session, and type of sports engaged in by the subjects were recorded. All subjects performed the one-legged stance test first, followed by the SEBT. Participants performed two test sessions 7 days apart. The first test was conducted by instructing participants to stand on one leg on a force plate (Advanced Mechanical Technology Inc. (AMTI, OR6-6, Massachusetts, USA)) and slightly flex the free leg at the hip and knee. The standing leg was slightly flexed at the knee with eyes open. Maintaining their hands on their waist, they focused on an imaginary object straight ahead. The testing protocol included 3 repetitions of 15 seconds for each leg. The starting limb was chosen randomly. After the examiner instructed and demonstrated the testing situation, participants were given one practice trial before the main test. Practice and test trials were considered invalid if the participant removed their hands from the waist, dropped down, or touched the force plate with the nonstanding limb, or moved the standing limb. Displacements of the COP in the mediolateral and anterior-posterior directions were recorded with Netforce (AMTI). The sampling frequency was 1000Hz and data was acquired for 15s. Time series signals were filtered using a Butterworth filter with a cut-off frequency of 12 Hz after which the following COP parameters were calculated for 10-second time interval: mean total COP displacement (COP_tot), mean displacement of the COP in anterior-posterior (COP_ap), and mediolateral (COP_ml) directions.

The SEBT was carried out after the one-legged stance test was completed. The shortened version includes the anterior, posteromedial, and posterolateral directions. 3 tape measures with a centimetre scale were affixed onto the laboratory floor. The first reach direction was aligned anterior to the apex; the other two were oriented 135 degrees to the first in the posteromedial and posterolateral directions [26]. Maintaining a single-leg stance, participants were instructed to reach out as far as possible with the nonstance limb along the marked tape, point to the most distal portion with their big toe, and return the limb back to the starting position [18]. Subjects practiced each direction 4 times before the actual testing to minimize learning effects [27, 28]. This was followed by the recording of 3 successful trials in each direction for both legs, always with a 10-s rest between each test [28]. The order of the starting limb was randomized, and the chronology of the directions was defined ((1) Anterior, (2) Posteromedial, and (3) Posterolateral). The subject's starting foot was placed on the convergence of the reach directional lines of the SEBT [26]. In this way, the lateral malleolus was positioned at the intersection point of the 3 directions, with the foot's longitudinal axis oriented towards the anterior direction. The starting position was a bilateral limb stance. Subjects performed the test with socks on and kept their hands on their hips throughout the testing period. The limb length of the subjects was then taken with a measuring tape. This was defined as the distance from the anterosuperior iliac spine to the medial malleolus [29]. Maximum reach distance was visually read by the same examiner for all subjects. A trial was considered invalid if the reaching foot did not return to the starting position, if it touched down whilst reaching out, if the support limb shifted, if the heel of the support foot did not stay in contact with the ground or if the hands were removed from the hips.

### 2.3. Outcome Measures and Statistical Analysis

Outcome measures of interest included the mean of the total COP displacement (COP_tot) and the mean displacement of the COP in the anterior-posterior (COP_ap) and mediolateral directions (COP_ml), all in millimetres. Mean normalized reach distance in anterior, posteriomedial, and posterolateral directions was expressed as the percentage of limb length and composite reach distance score (CRDS) for the SEBT [30]. The composite reach distance was calculated as the sum of the 3 normalized SEBT scores [30].

The relevant (nondigital) data for analysis was handwritten into a case report form, after which the computation was performed. Mean and standard deviations followed by paired t-tests and a Wilcoxon signed rank test for normally and nonnormally distributed data, respectively, were carried out. The intraclass correlation coefficient ICC (2, 1) for both limbs for each outcome measure was then calculated. Criteria ranges for ICC reliability were as follows: < 0.40, poor; 0.40 to 0.75, fair to good and > 0.75, excellent reliability [31]. Also, a Bland-Altman analysis [32] was used as an indicator of absolute reliability. The difference of the test-retest scores was plotted against their average as well as the limits of agreement. In addition to these, a post hoc power analysis was carried out using G Power 3.1.9.2 [33] to determine whether the research was adequately powered. The effect size (f) was calculated using the formula (mean of test —mean of retest)/pooled standard deviation of both tests. Bonferroni corrections were carried out to correct for any type-one error that might occur due to multiple analyses on the same dependent variable; hence the level of significance was set at *α* = (0.05/4) = 0.0125. Statistical analysis was carried out using SPSS version 24 (SPSS Inc., Chicago, IL, USA).

## 3. Results

The scores from the two testing sessions did not reveal any significant difference (P > 0.0125) in the outcome measures of the one-legged stance test for subjects with and without back pain as shown in Tables 1(a) and 1(b).

BP and NBP subjects recorded the ICCs of 0.54 to 0.69 and 0.45 to 0.60, respectively, for all the outcome measures of the one-legged stance test (Table 2).

There was no significant difference (p > 0.0125) in any of the directions for the SEBT between test-retest scores for both limbs of BP and NBP subjects, as reported in Tables 3(a) and 3(b).

ICCs of (0.75 to 0.93) and (0.60 to 0.85) were recorded for subjects with and without back pain, respectively, for the outcome measures of the SEBT as shown in Table 4.

**Figure 1:**
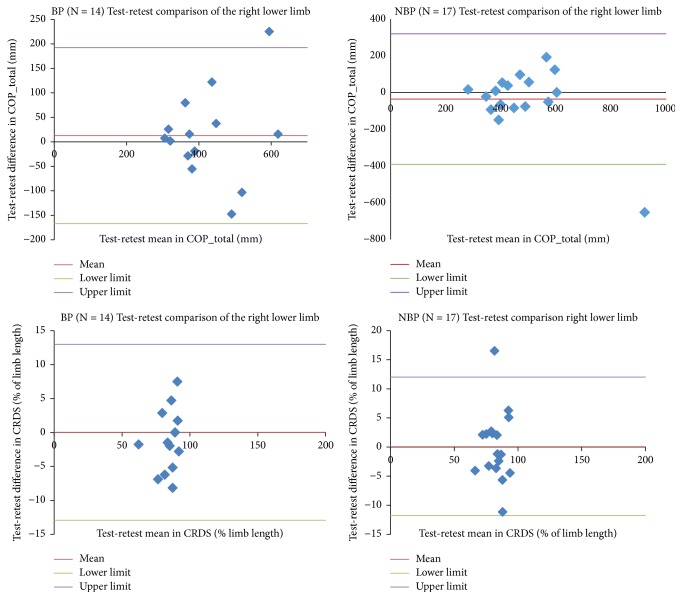
Bland-Altman plot for the right lower limb of adolescent athletes with and without back pain on the one-legged stance test and the star excursion balance test. Blue diamond: single values; mean – (bias); lower limit (bias – 1.96*∗*standard deviation); upper limit (bias + 1.96*∗*standard deviation).

Test-retest values did not reveal any significant difference (P > 0.0125) between the right and left lower limbs of athletes with and without back pain for all outcome measures of both the one-legged stance test and the SEBT when the 95% CIs are observed. Only results of COP_tot and CRDS of the right lower limb are reported for the Bland-Altman, as there was no significant difference or systematic bias between test and retest for subjects with and without back pain (Figure 1).

## 4. Discussion

The study aimed to determine the test-retest reproducibility of static and dynamic PC in adolescent athletes with and without back pain using the one-legged stance test and the SEBT. It also aimed to determine whether there was a difference in the reliabilities of the dynamic and static PC tests. The present results show that, in adolescent athletes with and without back pain, the reliability of the one-legged stance test is fair to good on all outcome measures. Also, the reliability of the SEBT is good to excellent for subjects with and without back pain. In addition to these, there was no statistically significant difference in the reliabilities of either the static or dynamic test for adolescent athletes with and without back pain.

The fair-to-good reliability of the outcome measures of the one-legged stance test for adolescents both with and without back pain adds to the various COP parameters reported to be reliable in literature [12–15, 34]. The results, however, cannot be directly compared without caution to those reported in the literature due to differences in the study population, testing duration, COP parameters used, type of stance employed, and testing surface used. The most reliable test-retest reliability was detected in female gymnasts whilst standing on a foam surface during 60s-test durations performing bipedal task [15]. This observation differs from the fair-to-good reliability observed in the current study using a test duration of 15s, a firm surface, and the one-legged stance test. This could be because postural control deficits become more evident during the execution of challenging tasks, as well as the need to challenge the postural control system in order to obtain useful information from the COP measurements [15] due of the study population. Thus, the one-legged stance test might have provided the needed challenge.

The total mean displacement of the COP in the present study was almost 3 times lower for both test and retest in back pain subjects compared to that obtained by Muehlbauer et al. [34] (test: 1,223.2 mm and 1,133.1 mm and retest: 1,099.3 mm and 1,013.3 mm for men and women, respectively). This difference could be due to the younger study population in the present study. Greater postural sway is reported in older compared to younger adults when the base of support is narrowed [35], and even more so with athletes who generally have superior balance ability due to participation in sports [36]. Also, the test-retest values recorded for back pain athletes were lower compared to those without back pain (Table 1) in the present study. This, however, did not reach a significant level, as observed in the 95% CIs. Harringe et al. [15] reported a nonsignificant difference between their back pain and healthy subjects, supporting the current results. This could be due to the adoption of alternative postural control strategies by the athletes with back pain to cope with the new demands introduced by the pain [7].

The reliability of the SEBT in adolescent athletes with back pain was excellent (ICC: 0.75 to 0.93), whilst that for those without pain was good to excellent (ICC: 0.60 to 0.85). These results are in the range of ICC values previously reported for healthy adults (0.67 to 0.87 [11], 0.78 to 0.96 [27]), basketball athletes (0.84 to 0.87) [19], and primary school children (0.51 to 0.93) [22]. The current investigation supports the reliability of the SEBT in adolescent athletes with and without back pain. The body relies on rapid, continuous feedback from three integrated but independent sensory sources to execute smooth and coordinated neuromuscular actions [37]. As back pain influences the trunk as well as lower limb movement [38], there is the possibility of detecting deficits in dynamic postural control using the measure of reach distance. This is because the feedback from the reach leg to the sensory sources of the postural control might be interrupted during performance of the SEBT [26]. Therefore, application of this tool in adolescent athletes may prove a more challenging task that could help further assess and monitor deficits resulting from back pain. A Bland-Altman plot for COP_tot showed little suggestion of a bias, as the mean differences between the test and retest of all the outcome measures for the one-legged stance test for athletes with and without back pain were close to zero. Muehlbauer et al. [34] also reported similar results for COP_tot in their investigation involving healthy adults on the one-legged stance test, supporting the current result. The good-to-excellent reliability reported for the SEBT was confirmed in the Bland-Altman analysis. Based on the plot, the conclusion can be drawn that there is no statistically significant difference between the test-retest scores of the outcome measures of the SEBT reported in this investigation. Bland-Altman analyses have not been reported in published investigations of reproducibility involving the SEBT; hence a direct comparison cannot be made with the published literature.

The confidence intervals of the reliabilities of the static and dynamic tests overlapped for subjects both with and without back pain. Also, within each test, there was an overlap of the confidence intervals between subjects with and without back pain. Therefore, one can conclude that there is no statistically significant difference between the static and dynamic tests, as well as between subjects with and without back pain, in our study population. A power analysis showed that, based on the lowest (f = 0.010) and highest (f = 0.527) effect sizes observed in the present study, approximately 95053 and 37 subjects would be needed respectively in both BP and NBP groups to obtain a statistical power at a 0.80 level [39].

### 4.1. Limitations of the Study

The pain questionnaire may be considered a limitation of this study, as it only assessed pain within 7 days prior to participation in the study. Hence, the possibility of varying phases and locations of BP and its effect on the current results cannot be ruled out. In addition to this, a mean pain score of 3.0 ± 0.8 for our cohort might be too low to show previously reported impact of back pain on postural control. Another limitation might be the varying sports disciplines considered together in the study, as the SEBT might be sensitive to sport-related adaptations [40], and the distinct skill requirements and environmental demands of different sports likely pose different challenges to the sensorimotor systems [41]. In addition to these, further investigation is required to ascertain the effect of gender on the current results, as there is a lack of agreement on the effect of gender on the SEBT, with the literature reporting both no effects [16, 31, 42] and significant effects [43, 44] after normalization. Another limitation could be the sample size since to produce studies that can detect clinically relevant differences the appropriate sample size has to be determined. However, based on the smallest and largest effect sizes, the sample size can be said to be within an appropriate range for the current study. All the same this should be taken into consideration when interpreting the results. Finally, many COP parameters are reported in the literature; therefore the choice of COP_tot, COP_ml, and COP_ap might not be enough to allow for a generalization of the results on the reproducibility of the one-legged stance test in adolescents with and without back pain.

## 5. Conclusion

Static and dynamic postural control test like the one-leg stance test and star excursion balance test show fair to excellent reliabilities in adolescent athletes with and without back pain. Based on the current study population there was no difference in the reliabilities between the healthy athletes and those with back pain.

## Figures and Tables

**Table:** (a) Mean ± SD (mm), 95% CI, and effect size for the outcome measures of the one-legged stance test for subjects with back pain during test and retest sessions.

		Test		Retest		

	Rt	Mean ± SD (mm)	95% CI	Mean ± SD (mm)	95%CI	Effect size

BP	COP_ap	295.8 ± 94.8	241.0 – 350.5	253.0 ± 64.5	215.8 – 290.3	0.527
	COP_ml	263.6 ± 58.8	229.6 – 297.5	274.2 ± 75.6	230.6 – 317.9	-0.157
	COP_total	434.6 ± 113.9	368.8 – 500.4	413.9 ± 104.1	353.8 – 474.0	0.190
	Lt					
	COP_ap	276.3 ± 100.2	218.4 – 334.2	268.7 ± 79.3	222.9 – 314.5	0.084
	COP_ml	253.9 ± 51.4	224.2 – 283.6	266.1 ± 75.1	222.7 – 309.5	-0.190
	COP_total	416.4 ± 113.5	350.9 - 481.9	424.7 ± 114.8	358.4 - 491.0	-0.059

left/right: Lt/Rt.

**Table:** (b) Mean ± SD (mm), 95% CI, and effect size for the outcome measures of the one-legged stance test for subjects without back pain during test and retest sessions.

		Test		Retest		

	Rt	Mean ± SD (mm)	95% CI	Mean ± SD (mm)	95%CI	Effect size

NBP	COP_ap	288.1 ± 72.1	251.0 – 325.1	315.1 ± 123.8	251.5 – 378.7	-0.238
	COP_ml	292.5 ± 82.4	250.1 – 334.8	307.5 ± 130.1	240.6 – 374.4	-0.121
	COP_total	462.5 ± 122.0	399.8 – 525.3	498.0 ± 211.3	389.3 – 606.7	-0.184
	Lt					
	COP_ap	288.8 ± 69.9	252.9 – 324.8	292.7 ± 80.5	251.3 – 334.1	-0.043
	COP_ml	296.6 ± 61.9	264.8 – 328.5	274.1 ± 67.9	239.1 – 309.0	0.287
	COP_total	459.0 ± 101.1	407.0 – 511.0	446.1 ± 112.3	388.3 – 503.8	0.100

left/right: Lt/Rt.

**Table 2:** Intraclass correlation coefficients with 95% confidence interval for the one-legged stance test calculated for testretest reliability for BP and NBP.

			Rt		Lt

		ICC	95% CI	ICC	95%CI

BP	COP_ap	0.69	0.3 – 0.9	0.65	0.2 – 0.9
	COP_ml	0.61	0.1 – 0.9	0.54	0.0 – 0.8
	COP_tot	0.63	0.2 – 0.9	0.64	0.2 – 0.9

NBP	COP_ap	0.49	0.0 – 0.8	0.52	0.1 – 0.8
	COP_ml	0.47	0.0 – 0.8	0.60	0.2 – 0.8
	COP_tot	0.45	-0.2 – 0.8	0.57	0.1 – 0.8

left/right: Lt/Rt.

**Table:** (a) Mean ± SD (% of limb length), 95% CI, and effect size for the different directions on the SEBT during test and retest for back pain subjects.

		Test		Retest		

	Rt (reach distance)	Mean ± SD (% of limb length)	95% CI	Mean ± SD (% of limb length)	95%CI	Effect size

BP	Anterior	89.3 ± 8.2	84.5 - 94.0	88.4 ± 6.2	84.8 – 91.9	0.097
	Posteromedial	83.4 ± 10.0	77.7 – 89.2	84.5 ± 8.4	79.7 – 89.3	-0.095
	Posterolateral	79.6 ± 9.4	74.2 – 85.0	81.9 ± 9.5	76.4 – 87.4	-0.199
	CRDS	84.1 ± 8.8	79.0 – 89.2	84.9 ± 7.6	80.5 – 89.3	-0.078
	Lt					
	Anterior	89.3 ± 7.4	85.0 – 93.6	89.0 ± 5.8	85.6 – 92.3	0.354
	Posteromedial	85.1 ± 10.1	79.2 – 90.9	84.7 ± 9.9	79.0 – 90.4	0.033
	Posterolateral	79.4 ± 10.0	73.6 – 85.2	81.8 ± 10.4	75.8 – 87.8	-0.193
	CRDS	84.6 ± 8.8	79.5 – 89.6	85.2 ± 8.4	80.3 – 90.0	-0.057

left/right: Lt/Rt.

**Table:** (b) Mean ± SD (% of limb length) 95% CI, and effect size for the different directions on the SEBT during test and retest for subjects without back pain.

		Test		Retest		

	Rt (reach distance)	Mean ± SD (% of limb length)	95% CI	Mean ± SD (% of limb length)	95%CI	Effect size

NBP	Anterior	88.4 ± 8.2	84.2 – 92.6	87.8 ± 7.8	83.8 – 91.8	0.061
	Posteromedial	79.9 ± 9.2	75.2 – 84.6	81.0 ± 9.3	76.3 – 85.8	-0.097
	Posterolateral	79.8 ± 9.3	75.0 – 84.6	78.9 ± 9.8	73.9 – 84.0	0.078
	CRDS	82.7 ± 7.9	78.6 – 86.6	82.6 ± 8.0	78.5 – 86.7	0.010
	Lt					
	Anterior	88.9 ± 8.6	84.5 – 93.4	89.2 ± 7.5	85.4 – 93.1	-0.030
	Posteromedial	80.6 ± 10.4	75.3 – 86.0	81.2 ± 10.0	76.1 – 86.4	-0.048
	Posterolateral	78.1 ± 12.6	71.6 – 84.6	78.6 ± 10.0	73.5 – 83.7	-0.035
	CRDS	82.6 ± 9.7	77.6 – 87.6	83.0 ± 8.3	78.7 – 87.3	-0.035

left/right: Lt/Rt.

**Table 4:** Intraclass correlation coefficients with 95% confidence interval for test-retest reliability of subjects with and without back pain on the SEBT.

Test-retest reliability					

			Rt		Lt

	Reach distance (% of limb length)	ICC	95% CI	ICC	95% CI
BP	Anterior	0.79	0.4 – 0.9	0.75	0.4 – 0.9
	Posteromedial	0.88	0.7 – 1.0	0.89	0.7 – 1.0
	Posterolateral	0.85	0.6 – 0.9	0.93	0.8 – 1.0
	CRDS	0.86	0.6 – 1.0	0.91	0.7 – 1.0

NBP	Anterior	0.82	0.6 – 0.9	0.85	0.6 – 0.9
	Posteromedial	0.79	0.5 – 0.9	0.60	0.2 – 0.8
	Posterolateral	0.61	0.2 – 0.8	0.65	0.3 – 0.9
	CRDS	0.74	0.4 – 0.9	0.69	0.3 – 0.9

left/right: Lt/Rt.

## Data Availability

The data for the current study are available from the authors on reasonable request.
